# A Data-Gathering, Dynamic Duty-Cycling MAC Protocol for Large-Scale Wireless Sensor Networks

**DOI:** 10.3390/s20154071

**Published:** 2020-07-22

**Authors:** Fei Tong, Yuyang Peng

**Affiliations:** 1 School of Cyber Science and Engineering, Southeast University, Nanjing 210096, China; 2 Faculty of Information Technology, Macau University of Science and Technology, Taipa 999078, Macau, China; yypeng@must.edu.mo

**Keywords:** MAC, routing, duty cycle, data-gathering, wireless sensor networks

## Abstract

This paper presents a Data-gathering, Dynamic Duty-cycling (D3) protocol for wireless sensor networks. With a proposed duty-cycling MAC of high energy efficiency in D3, a routing scheme is naturally embedded to reduce protocol overhead. A packet can be forwarded in a pipelined fashion by staggering the sleep-wakeup schedules between two communicating nodes, which can significantly reduce end-to-end delay to meet real-time transmission requirements. To construct and maintain schedules, a grade and schedule establishment mechanism with a lightweight schedule error correction scheme is designed. In addition, based on the intrinsic characteristics of the network, an adaptive schedule maintenance scheme is proposed to dynamically adjust the node duty cycle to the network traffic load. The results based on the extensive OPNET simulations show that D3 can largely improve packet delivery ratio, energy efficiency and throughput, and reduce packet delivery latency.

## 1. Introduction

Different applications of Wireless Sensor Networks (WSNs) have different topology requirements and communication paradigms, and thus require different network design approaches [1]. One of their significant applications is long-term sustainable monitoring, such as natural environment [2] and structure-monitoring [3,4], industrial process monitoring [5], etc. In such scenarios, a number of sensors are deployed either randomly or in a specific topology (e.g., linear topology) with one or a few sink nodes, where the sensed data are forwarded to the sink node in a many-to-one communication paradigm.

It is crucial for WSNs to utilize energy efficiently because of the limited capacity of the battery equipped by sensors. A series of MAC protocols were proposed [6,7,8,9,10,11] to adopt the duty-cycling mechanism for energy conservation. Using this mechanism, sensor nodes alternately wake up and sleep based on their schedules. Thus the energy efficiency of a WSN can be improved by reducing the two of the most principal sources of energy wastage in WSNs, i.e., idle listening [12] and overhearing [13].

However, the duty-cycling mechanism introduces a significant rise in packet delivery latency, known as sleep latency [14,15], which makes itself not helpful for delay sensitive monitoring. Specifically, a packet can only be sent out when the transmitter and receiver rendezvous with each other in their awake state. The problem becomes more serious in the multi-hop communications as the data forwarding process is interrupted if the next forwarding node is asleep, accumulating to a large end-to-end (E2E) packet delivery latency. Although some of the existing work proposed the pipeline forwarding schemes to alleviate this problem [12,16], they were designed independently under the pure scheduling perspective without considering other issues potentially facing a WSN, such as the joint study involving the MAC, routing, schedule synchronization issues, etc.

Another issue is that the conventional duty-cycling MAC protocols usually adopt a fixed duty cycle for energy conservation, which is not desirable in a network with dynamic traffic load. A duty cycle for high traffic load will cause a significant waste of energy when the traffic load becomes low, while a duty cycle for low traffic load will result in a long packet delivery latency. Therefore, an energy-efficient duty-cycling mechanism is expected to adapt itself dynamically.

In addition, due to the resources limit, the design of MAC and routing protocols for WSNs are expected to be seamlessly integrated, while most of the conventional MAC protocols have no consideration about the routing issue. The incorporation of an independent routing protocol to a MAC, however, increases the protocol overhead and traffic load, leading to degraded network performance and reduced network lifetime.

Furthermore, to eliminate the adverse impacts of clock drift and hardware/operating system latency, most prior duty-cycling MAC protocols [6,17,18,19] depend on underlying support of a time synchronization mechanism. They assume that a separate synchronization protocol [20,21,22,23] works well with the required precision. However, the synchronization itself could be costly and difficult to implement in a duty-cycling network due to the lack of broadcasting capability in WSNs for periodic time-stamp exchanges during synchronization [24].

This paper proposes a data-gathering, dynamic duty-cycling (D3) protocol by jointly studying the problems listed above. In D3, routing functionality is embedded to reduce the network communication overhead, and the superiority of the duty-cycling mechanism is kept to increase the energy utilization efficiency. Those nodes along the path from a source node to the sink have staggered schedules, and thus the packet can be forwarded in a pipelined fashion, largely reducing the packet delivery latency and efficiently handling the traffic contention by moving traffic quickly away from the contention area. Each node only maintains, in total, three attributes including node grade, current state, and current state duration, which improves the scalability with respect to network topology changes. Moreover, D3 intrinsically supports multiple sinks [25] to partition a large-scale WSN into several independent subnetworks, which increases the network manageability and balances the energy dissipation. Other main contributions of this paper include the following:

- With the Adaptive Schedule Maintenance (ASM) scheme, D3 allows a node to utilize its sleeping slots on demand according to the network traffic load, and thus the duty cycle can be dynamically adjusted, which can largely improve the network energy efficiency.
- A lightweight schedule error correction scheme achieves 0-bit synchronization as in [24], by extracting the information naturally embedded in the received packets.
- Meanwhile, D3 has been implemented in OPNET, which is a well-known, industry-strength network simulator with high simulation fidelity. Extensive OPNET simulations are conducted for performance evaluation in comparison with existing work.

The rest of the paper is organized as follows. Section 2 briefly surveys the related work. Section 3 presents the design of D3 in detail, including the network scalability discussion. Section 4 presents the performance evaluation based on OPNET simulations. Finally, Section 5 concludes the paper.

## 2. Related Work

Existing duty-cycling MAC protocols can be categorized into two classes: asynchronous [8,9,10,26,27,28,29] and synchronous [6,7,25,30,31,32,33]. The asynchronous designs allow each node to establish and maintain its own schedule independently. For example, the authors in [10] proposed PW-MAC, one of the state-of-the-art asynchronous duty-cycling MAC protocols, for example. It alleviates the energy-efficiency problem by utilizing an independently generated pseudo-random sequence to allow a sender to accurately predict its receiver’s wakeup time. Thus, a sender can reduce its duty cycle by waking up right before the receiver wakes up. Although PW-MAC is energy-efficient, it introduces packet delivery latency and handles traffic contention inefficiently, as a packet can only be forwarded in 1-hop distance in each operational cycle. In particular, the replying ACK from a receiver may collide with those beacons broadcast by other nodes who just wake up for receiving potential packets. It leads to unnecessary retransmission since the packet actually has been received successfully. In [26], the authors proposed a cooperative asynchronous MAC protocol, called COASYM-MAC, based on a receiver-initiated MAC. In [29], the authors proposed PAX-MAC, a preamble ahead cross-layer MAC, which is designed for low latency packet propagation in duty-cycled WSNs. It consists of two phases, including the asynchronous propagation of preambles to establish a route and the packet forwarding along the established route. Overall, the existing asynchronous MAC protocols eliminate the synchronization overhead, however, by sacrificing (1) energy efficiency, since a transmitter (receiver) has to wake up much earlier to rendezvous with its receiver (transmitter), (2) channel efficiency with high collision possibility and unnecessary retransmission, and (3) E2E delay in multi-hop-transmission scenarios.

On the contrary, the synchronous designs require neighboring nodes to be time-synchronized. The solutions improve channel efficiency because the nodes’ sleep-wakeup schedules can be controlled and tracked. However, it could still suffer significant E2E delivery latency due to the same reason as in the asynchronous MAC protocols; and underlying support from an independent synchronization protocol is needed, which is costly and difficult to implement since the duty-cycling network is lack of broadcasting capability for periodic time-stamp exchanges [24]. In our previous work, P-MAC [25] was proposed to solve the first problem by allowing pipelined packet forwarding, which is widely utilized in the pure scheduling schemes without considering the MAC issues [12,16]. In [31], the authors proposed a traffic-adaptive synchronous MAC (TAS-MAC) for WSNs. The TDMA mechanism is adapted to a traffic-adaptive allocation mechanism to achieve high throughput. In addition, by notifying the nodes on active routes of incoming traffic in advance, the E2E delay can be reduced. For the second problem, a scheme with 0-bit synchronization [24] is inherently achieved in this paper.

Both the conventional asynchronous and synchronous duty-cycling protocols were designed independently without considering routing. The addition of a routing protocol would cause a significant performance degradation due to the increase of protocol overhead. In addition, the node identification issue was also ignored, which suggests that these protocols are not suitable for a large-scale WSN. Moreover, they are only applicable in stable traffic conditions because of their fixed duty cycles. Due to the burst traffic load caused by converge-cast, correlated events or data aggregation [11], however, a fixed duty cycle will degrade the performance of these protocols, especially in the network with a varying traffic load.

## 3. Protocol Design

The design overview of D3 is first introduced in Section 3.1. Then, Section 3.2 introduces node identification in D3, Section 3.3 presents the grade and schedule establishment scheme, and Section 3.4 presents the lightweight schedule error correction mechanism. Finally, Section 3.5 shows the adaptive schedule maintenance mechanism.

### 3.1. Design Overview

In D3, each node is assigned to a grade, which is the minimum communication hop distance to the sink node [34]. The sink node is in grade zero and initializes this grade division process by broadcasting a DIVISION message. The grade of each node will be utilized for data transmission, as sensed data can be forwarded towards the sink from the higher to lower grades. The node transmission range is modeled as an ideal circle. The network is divided into grades similar to concentric circles with the sink node located at the center.

As long as a node determines its grade, it simultaneously sets its periodical sleep-wakeup schedule. The two schedules in two adjacent-grade nodes, respectively, are staggered so that a pipeline for data propagation is created to reduce the packet delivery latency. The above Grade and Schedule Establishment (GSE) will be introduced in detail in Section 3.3. With this mechanism, D3 can effectively support multiple sinks to increase the network manageability and balance the energy dissipation [25]. A node in grade *i* (i>0) with a pipelined schedule periodically experiences three states: (1) **R**: receiving a data packet from a node in grade i+1; (2) **T**: transmitting a data packet to a node in grade i−1; and (3) **S**: sleeping. Thus, after receiving a data packet during its **R** state, the node can forward it during its coming **T** state without sleep latency.

**Figure 1:**
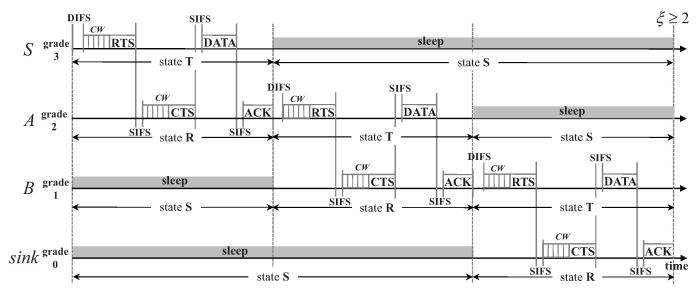
Data transmission process in D3 with the CW2-RTS/CTS handshake; node *S* sends data to the sink node.

Since a sensor node with pending data is not aware of the routing path to the sink node, an RTS/CTS-like handshake (denoted by CW2-RTS/CTS) mechanism is proposed for it to determine the next-hop node from its adjacent lower-grade nodes. The proposed handshake mechanism differs from the original RTS/CTS handshake adopted by IEEE 802.11 in three aspects. First, in D3, the grade information of a source node is embedded in its RTS packet. After those nodes in the adjacent lower grade receive this RTS packet, they will contend for replying with CTS. Second, for each data transmission attempt, each node uses a randomly-generated identifier (RID) as the node address instead of a pre-allocated MAC address. Therefore, the RTS packet broadcast by each source node contains an RID, and so does the CTS replied from the lower-grade nodes. This node identification scheme will be introduced in detail in Section 3.2. Third, since there also exists contention among the adjacent lower-grade nodes of each source node when they reply with CTS, a Contention Window (CW) is also set for them, as shown in Figure 1. The format of the RTS/CTS packet is shown in Table 1.

When a node enters state **T**, its upper-grade nodes and the lower-grade nodes of its adjacent lower-grade nodes are all in state **S**, which helps to avoid channel contention, data collision and overhearing. On the other hand, those nodes in the same grade with pending data contend for a channel with each other by broadcasting an RTS in state **T**. Before sending its own RTS, if a node overhears an RTS from another node in the same grade, it will enter the **S** state immediately. In addition, it is possible that there are multiple nodes in the same grade receiving the same RTS in the **R** state, then they also contend with each other for replying with a CTS. Similarly, if a node overhears a CTS from another node in the same grade, it will enter the **S** state immediately.

Take the network shown in Figure 1 for example. Suppose node *S* is the source, and it wins the medium in its **T** state after contending with its neighboring nodes (e.g., node $S^{\prime }$) in the same grade and then broadcasts an RTS packet containing an RID. These neighboring nodes will enter state **S** to avoid packet collision and overhearing for saving energy, if they overhear the RTS packet sent by *S*. Suppose that both *A* and $A^{\prime }$ receive this RTS in their **R** state. Then they contend with each other for replying with CTS embedded with their own RIDs. If it is the node *A*’s CTS that is first received by node *S*, then *S* sends its data packet to *A*. Similarly, for the neighboring nodes of *A* in the same grade (e.g., node $A^{\prime }$), they will enter state **S** if they overhear the CTS packet from *A*. After receiving the data packet from *S*, *A* will reply with an ACK packet and enter its **T** state. *S* enters state **S** after receiving the ACK packet from *A*. Node *A* in state **T** will determine its relaying node and send data packet in the same way. The aforementioned data transmission process is shown in Figure 1. With the routing functionality naturally embedded in the handshake process, the protocol overhead is largely reduced.

As shown in Figure 1, the duration of **T** is equal to that of **R**, which is viewed as a slot for one packet transmission. The maximum duration of a slot is

(1) $$T_{slot}=2W\sigma +DIFS+3SIFS+\mathrm{durRTS}+\mathrm{durCTS}+\mathrm{durDATA}+\mathrm{durACK}\;,$$

where *W* is the maximum number of time mini-slots in CW, σ is the time duration of each mini-slot, and durRTS, durCTS, durDATA and durACK are the one-hop transmission duration of the RTS, CTS, DATA and ACK packets, respectively. For pipeline scheduling, the duration of the **S** state must be an integer multiple of a slot duration, which is

(2) $$T_{S}=\xi \cdot T_{slot}\;,$$

where the positive integer ξ is referred to as sleep factor. Therefore, the whole cycle duration is $T_{cycle}=\left(\xi +2\right)\cdot T_{slot}$.

If ξ=1, when the *i*th-grade (i>0) nodes enter the **R** state, the (i−1)th-grade nodes are still in state **S**, and the (i+2)th-grade nodes just enter and will stay in the **S** state for at least $\xi \cdot T_{\mathrm{slot}}=T_{\mathrm{slot}}$ time. Therefore, for the communications between the *i*-th- and the (i+1)-th-grade nodes, there would be no interference coming from the (i−1)-th- and (i+2)-th-grade nodes. Considering the interference range is about twice the transmission range [35], ξ needs to be set to at least 2 as shown in Figure 1. Thus node *S* and those nodes in the grades farther away will not interfere with the communication between *B* and sink.

### 3.2. Node Identification

**Figure 2:**
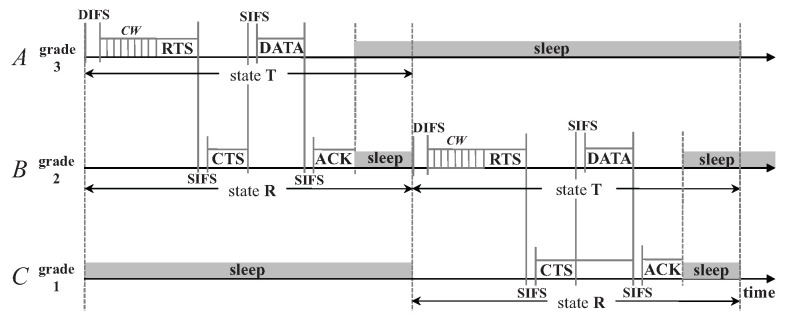
CW-RTS/CTS: node *A* has node *B*’s RID in its Next Hop table, so its RTS is dedicated for node *B* and node *B* replies with CTS without contention.

To avoid the cost of assigning a unique address to each sensor node during the manufacturing phase and the overhead of performing an independent address allocation and exchange mechanism at runtime, a node in D3 will randomly generate its identifier when it is involved in the packet transmission. In the early stage of the network, a receiver has to contend for replying with a CTS with its RID embedded after receiving an RTS from its sender, since the sender has no idea of its next-hop node if no independent routing protocol is performed beforehand (see Figure 1). When a node successfully transmits/receives a packet, it keeps the new RID as its identifier for the following packet transmissions. Meanwhile, during a successful transmission, the sender keeps a record of the receiver’s RID in its Next Hop table. In the following transmissions, the sender will select its next hop from this table either randomly or preferentially according to a predefined pattern. In this way, the probability of a successful transmission can be largely improved by eliminating the reply contention of CTS (see Figure 2). Such a transmission is denoted by CW-RTS/CTS, in contrast to CW2-RTS/CTS as shown in Figure 1.

A node can learn the RIDs of its neighbors in the same grade and record these RIDs in its Neighbor Node table by overhearing the packets sent from them. Each node who has not determined its identifier will have a lookup of this table to avoid generating an RID which has already existed in the table. In this way, it ensures that those nodes in the same grade within the coverage of each other will not have identical RIDs, which in turn eliminates the ambiguity of node identification during the data transmission. Notice that it does not matter that those nodes in different grades have identical RIDs, since they can differentiate from each other using their grade information.

### 3.3. Grade and Schedule Establishment (GSE)

This subsection introduces GSE in detail, which is utilized for grade division, and schedule construction and maintenance. In GSE, there are three attributes maintained by each node: (1) grade, with initial value of −1; (2) state, the current state of the node, which is in {**R**,**T**,**S**}; and (3) stateDur, the duration that the node has stayed for in the current state. Correspondingly, the DIVISION message contains three fields (as summarized in Table 2): (1) MGrade, the grade of the node sending the DIVISION message; (2) MState, the current state of the sending node; (3) MStateDur, the duration of the current state right before sending the message.

With the three attributes, i.e., grade, state, and stateDur, set to zero, **R**, and zero, respectively, the sink generates a DIVISION message with the three fields mentioned above set to the same values, i.e., zero, **R**, and zero, and then broadcasts it. Unless it has already identified an equal or a lower grade, a node receiving a DIVISION message first sets its attributes and pipelined schedule, updates the fields of the message, and then rebroadcasts the message.

**Figure 3:**
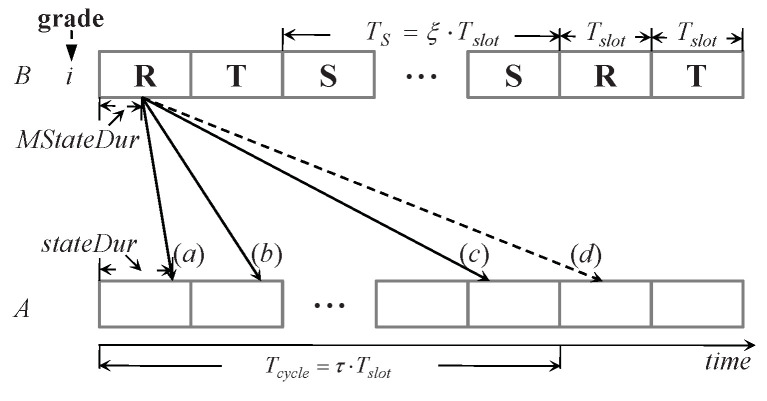
Cases (*a*), (*b*), and (*c*): node *A* receives a DIVISION message from node *B* in the current cycle; case (*d*): it receives a message in the next cycle.

Figure 3 shows an example for illustrating how a node processes a received DIVISION message. Node *B* has joined a grade *i*. Node *A* is within the transmission range of node *B* and has yet to join a grade or its attribute grade >i+1. After receiving a DIVISION message with MGrade=i, it sets its grade to i+1. The detail of how to set its other two attributes (i.e., state and stateDur) and schedule is shown as follows.

Suppose node *B* is currently in state **R** as shown in Figure 3 (the way is the same if *B* is in state **T** or **S**). Due to the message delivery latency (denoted by $T_{M}$, the difference between the message receiving time and generation time), the moment when node *A* receives the DIVISION message will be in state **T** but might also be in state **S** or **R**, corresponding to the case (*a*), (*b*) and (*c*) shown in Figure 3, respectively. If $T_{M}$ is long enough such that $\left\lfloor \frac{MStateDur+T_{M}}{T_{cycle}}\right\rfloor \geq 1$, node *A* will receive the message after one or more cycles, e.g., case (*d*) in the figure. According to the periodicity of the schedule, the following calculation is used to confine the set of *A*’s state and stateDur to three cases in one cycle: $t=MStateDur+T_{M}-\left\lfloor \frac{MStateDur+T_{M}}{T_{cycle}}\right\rfloor \cdot T_{cycle}$. If node *B* is currently in state **R** as shown in Figure 3, there are three cases for node *A*:

- case (*a*): $t<T_{slot}$. Node *A* should set its state to **T** and its stateDur to *t*. After ($T_{slot}-stateDur$) time, *A* enters the next state, **S**. If $\lfloor \frac{MStateDur+T_{M}}{T_{cycle}}\rfloor =1$, this is case (*d*) in Figure 3.
- case (*b*): $t<(T_{slot}+\xi \cdot T_{slot})$. Node *A* has transferred to state **S** from **T**. Node *A* will set its state to **S** and its stateDur to ($t-T_{slot})$. After ($T_{S}-stateDur$) time, it enters its next state, **R**.
- case (*c*): $t\geq (T_{slot}+\xi \cdot T_{slot})$. Node *A* will set its state to **R** and its stateDur to ($t-(T_{slot}+\xi \cdot T_{slot}))$. After ($T_{slot}-stateDur$) time, it enters the next state, **T**.

Node *A* can compute the $T_{M}$ of a DIVISION message as the difference between its local time when it receives this message and the message generation time embedded in the message. The algorithm used in GSE is summarized in Algorithm 1.
**Algorithm 1** GSE: processing a received DIVISION message1:$t\leftarrow MStateDur+T_{M}-\left\lfloor \frac{MStateDur+T_{M}}{T_{cycle}}\right\rfloor \cdot T_{cycle}$;2:**if**grade<0**or**grade>MGrade+1**then**3:    grade←MGrade+1;4:    **if** MState==R **then**5:        **if** $t<T_{slot}$ **then**6:           state←T;stateDur←t;7:        **else if** $t<\left(\xi +1\right)\cdot T_{slot}$ **then**8:           $\mathrm{state}\leftarrow S;\;\mathrm{stateDur}\leftarrow t-T_{slot}$;9:        **else**10:           $\mathrm{state}\leftarrow R;\;\mathrm{stateDur}\leftarrow t-\left(\xi +1\right)\cdot T_{slot}$;11:        **end if**12:    **end if**13:    **if** MState==T **then**14:        **if** $t<\xi \cdot T_{slot}$ **then**15:           state←S;stateDur←t;16:        **else if** $t<\left(\xi +1\right)\cdot T_{slot}$ **then**17:           $\mathrm{state}\leftarrow R;\;\mathrm{stateDur}\leftarrow t-\xi \cdot T_{slot}$;18:        **else**19:           $\mathrm{state}\leftarrow T;\;\mathrm{stateDur}\leftarrow t-\left(\xi +1\right)\cdot T_{slot}$;20:        **end if**21:    **end if**22:    **if** MState==S **then**23:        **if** $t<\left(\xi -1\right)\cdot T_{slot}$ **then**24:           $\mathrm{state}\leftarrow S;\;\mathrm{stateDur}\leftarrow t+T_{slot}$;25:        **else if** $t<\xi \cdot T_{slot}$ **then**26:           $\mathrm{state}\leftarrow R;\;\mathrm{stateDur}\leftarrow t-\left(\xi -1\right)\cdot T_{slot}$;27:        **else if** $t<\left(\xi +1\right)\cdot T_{slot}$ **then**28:           $\mathrm{state}\leftarrow T;\;\mathrm{stateDur}\leftarrow t-\xi \cdot T_{slot}$;29:        **else**30:           $\mathrm{state}\leftarrow S;\;\mathrm{stateDur}\leftarrow t-\left(\xi +1\right)\cdot T_{slot}$;31:        **end if**32:    **end if**33:    MGrade←grade;MState←state;MStateDur←stateDur;34:    rebroadcast the DIVISION message;35:**else**36:    discard the DIVISION message;37:**end if**

### 3.4. Schedule Error Correction (SEC)

The wakeup schedule error of a node is defined as the difference of the actual wakeup time and the scheduled wakeup time. Such an error is usually caused by unpredictable hardware/operating system latency and clock drift. Correcting schedule error is an important issue, because if a node wakes up much earlier or later than the scheduled wakeup time, it will miss the wakeup of its sender/receiver, leading to the failure of the current packet transmission and prolonging the packet delivery latency.

The Schedule Error Correction (SEC) in D3 is achieved by extracting the sending node’s three attributes and the packet generation time embedded in the received/overheard packets, which is referred to as 0-bit synchronization [24]. Therefore, the receiving node can adjust its schedule without periodic time-stamp exchanges by adopting the same mechanism as that used in the GSE process. Specifically, after finishing state **R**, a node with pending data needs to predict the time when its lower-grade nodes will wake up, and thus it can enter state **T** to start the packet transmission. In other words, the node will adjust the **T** schedule according to the **R** schedule of its lower-grade nodes.

**Figure 4:**
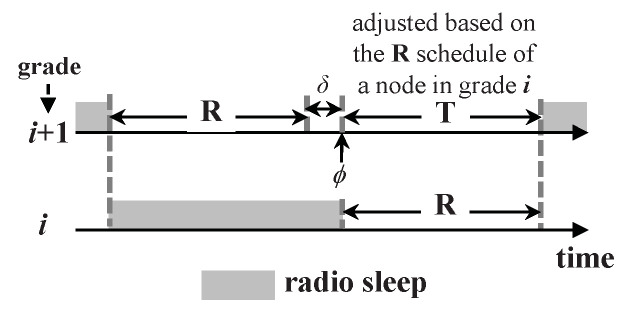
Guard time δ between **R** and **T**.

To ensure that the adjustment of a node’s **T** schedule will not affect its **R** schedule, there should be a guard time (denoted as δ) between **R** and **T** in each cycle, as shown in Figure 4. According to the experiment results based on MICAz motes in [10], δ reflects the schedule error variance caused by the clock drift and hardware/operating system latency. If the beginning time of **T** falls in [ϕ−δ,ϕ+δ] after a SEC process (where ϕ is the ending time of **R** plus δ), the schedule of **R** does not need to be adjusted; otherwise, it should be adjusted so that its ending time is equal to the current beginning time of **T** minus δ to guarantee the guard time.

A node can hear more than one lower-grade node in the same grade who may not exactly have the same schedule due to the unpredictable timing issues. It will make the node have different **T** schedules due to the different **R** schedules from these lower-grade nodes. The node can either store each obtained schedule corresponding to each lower-grade node in its Next Hop table, or adjust its **T** state long enough to contain each obtained schedule.

Each time when a node receives/overhears a packet from its lower-grade nodes, it determines whether to adjust its schedule by executing the SEC process. However, it is possible that the network can be entirely or locally idle so that a node could not receive any messages from its neighbor nodes. If this situation lasts for a pre-assigned time period, the node will keep awake to listen to the channel for a potential packet. A node that has not been involved in any transmissions but can overhear the packets from its lower-grade nodes to adjust its schedule, will broadcast a DIVISION message in its **R** state. Thus its higher-grade nodes can adjust their schedules based on the received DIVISION message.

Assume that each node in the network can reach the sink directly if the sink is within its transmission range, or via the relaying nodes in between. Through the mechanism mentioned above, a node can always adjust its schedule by receiving packets from its lower-grade nodes. It is obviously true when the node is involved in a packet transmission process. Let us investigate the cases when the network is entirely or locally idle.

Case 1: The network becomes entirely idle. After a pre-defined threshold of time, all nodes in the network will keep awake, and the sink node will broadcast a GRADE packet. This is known as the GSE process, after which the schedule for each node in the network can be reconstructed.

Case 2: The network is locally idle. If a node in grade *i* (i>1) is involved in packet transmissions, its lower-grade nodes must be involved in the transmissions, since they relay packets towards the sink. Therefore, only its upper-grade nodes are possibly idle—there are two cases: (1) if a node is idle in its **R** state but transmitting in its **T** state, then its upper-grade nodes can start the SEC process after overhearing an RTS/DATA packet; (2) if the node is completely idle with correct schedule adjusted by overhearing the RTS/DATA/GRADE packet from its lower-grade nodes, it will broadcast a GRADE packet towards its upper-grade nodes after a threshold of time.

### 3.5. Adaptive Schedule Maintenance (ASM)

The sleep factor ξ determines how long a node can sleep during a cycle. The larger the sleep factor, the lower the duty cycle and correspondingly the more the energy that can be saved. A longer sleeping time, however, will cause the packets queued in the buffer to wait longer, correspondingly leading to a large packet latency and low throughput. This situation becomes more serious when the network traffic load becomes heavier. This subsection shows the Adaptive Schedule Maintenance (ASM) scheme, which utilizes the node sleeping slots on demand according to the network traffic load, and thus the duty cycle can be adaptively adjusted, which can largely improve the network energy efficiency.

Specifically, during state **T**, a sender with more than one packet in its buffer will set a rendezvous flag in its RTS packet to inform its receiver of waking up to receive another packet during state **S**. If the grade of the receiver is larger than one, the receiver will also set a rendezvous flag during its coming **T** state, and thus the data can still be forwarded in a pipelined fashion. Next, the paper discusses such a non-last-hop transmission, separately from the last-hop transmission which is from the grade-one nodes to the sink.

#### 3.5.1. Non-Last-Hop Transmission, NLHT

**Figure 5:**
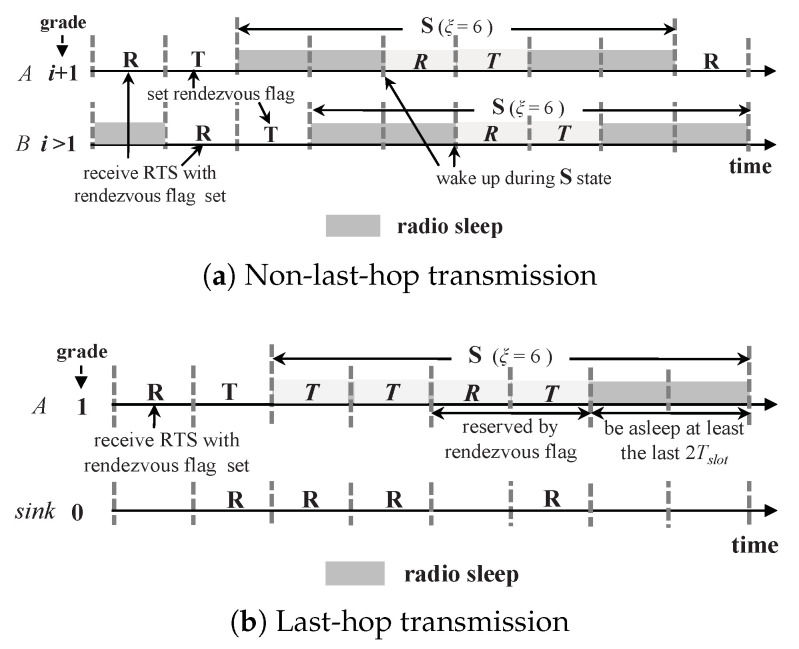
Adaptive Schedule Maintenance (ASM) scheme, for (**a**) Non-Last-Hop Transmission (NLHT) and (**b**) LHT.

Figure 5a shows an example for illustration, where node *A* in grade i+1 is communicating with *B* in grade i(i>1). Node *A* receiving an RTS in state **R** with the rendezvous flag set will also set this flag in its own RTS during its coming **T** state and adjust its schedule. So does node *B*. To avoid interfering with the communications in the lower-grade nodes, after a pair of **R** and **T**, a node should sleep at least two slots (2$T_{slot}$) and then wake up again. Therefore, to utilize sleeping slot for data transmission, ξ should be set to at least 6, and

(3) $$WT_{max}=\left\lfloor \frac{\xi -2}{4}\right\rfloor \;,$$

where $WT_{max}$ is the maximum slots a node can wake up during its **S** state for data transmission.

#### 3.5.2. Last-Hop Transmission, LHT

The grade-one nodes, which are heavily involved in relaying packets from their upper-grade nodes, are usually the bottleneck in data-gathering sensor networks [7,36]. In LHT, after receiving an RTS with the rendezvous flag set, a node in grade one does not need to set its own flag to inform the sink node of waking up, since the sink node is always awake for receiving packets. It only adjusts its schedule to wake up in state **S** for receiving a packet from its upper-grade sender. In addition, to avoid interfering with the communications in the upper-grade nodes, the grade-one nodes should sleep during at least the last two slots (2$T_{slot}$) of the **S** state; while the other slots in **S** can be utilized for data transmission except those reserved by the rendezvous flag. An example is shown in Figure 5.

Based on the above schemes, ASM can adaptively adjust the node duty cycle to the network traffic load by utilizing sleeping slots on demand. ξ determines the number of sleeping slots ASM can utilize. If the network traffic load is low, the regular **T** and **R** slots are enough for data delivery; while with a high traffic load, the sleeping slots will be utilized.

### 3.6. Scalability Discussion

With the above design, D3 can, not only reduce protocol overhead, but also can enhance network scalability:

- If a new node is added to the network, it will identify which grade it should join and the corresponding schedule it should choose after listening to its neighbors for a while. Subsequently, it can join the network by carrying out the GSE mechanism.
- It is relatively easy for D3 to support node mobility. A mobile node needs to redefine its grade and schedule if it moves out of its original grade area. Similar to a newly added node, it can rejoin the network later, unless it moves out of the network and thus becomes isolated.
- In a large-scale WSN, multiple sinks are needed to increase network manageability and balance energy dissipation. D3 can partition the whole network into several sub-networks by adopting its GSE mechanism. Each sink serves one sub-network independently.

## 4. Performance Evaluation

**Figure 6:**
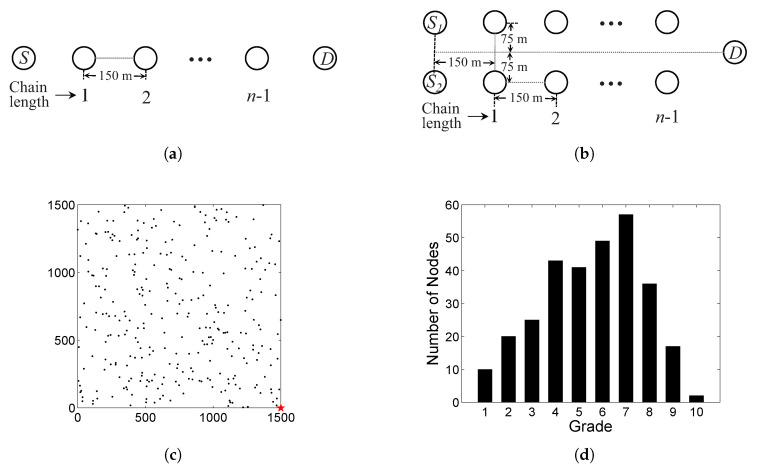
Network scenarios in simulations. (**a**) Single-chain network. (**b**) Double-chain network. (**c**) 2-D random network. (**d**) Grade distribution in the network after the GSE process.

In this section, the performance of D3 is evaluated by extensive OPNET simulations. The performance of D3 is compared with a basic duty-cycling and pipelined-forwarding protocol as the benchmark [37,38]. The comparison is based on two chain networks, namely, single-chain (Figure 6a) and double-chain (Figure 6b). The double-chain network with two sources is used to evaluate the protocol performance in the case of serious contention and interference, by comparing it in the case of the single-chain network with only one source. Finally, the performance evaluation is extended to a 2D random network. The above three network scenarios are shown in Figure 6. Each sensor node in the network is equipped an omnidirectional antenna, employing a combined free space and two-ray ground reflection radio propagation models. The network parameters are shown in Table 3. In particular, a set of the typical power consumption parameters for Mica2 radio (CC1000) are utilized for energy consumption statistics [39].

In both chain networks, the source node(s) will send packets to the destination node D with the nodes in chain(s) as relay nodes. The chain length varies from 0 to n−1. The maximum *n* is set to 20. Therefore, the path length from *S* to *D* varies from 1–20 (n= 1–20) hops. When n=1, *S* sends packets directly to D. The 2D random network is shown in Figure 6c. The network consists of 300 sensor nodes randomly distributed in a 1500 × 1500 m${}^{2}$ square area, and a sink located at the bottom-right corner of the square shown as a star in the figure. Figure 6d shows the corresponding grade distribution in the 2D random network after the GSE process. The maximum grade is 10, and most of sensor nodes are in grade 7. The traffic in the network is generated as follows: a random node is periodically selected to send a packet, which simulates a generated event following a Poisson distribution.

### 4.1. Performance Evaluation in the Chain Network Scenario

**Figure 7:**
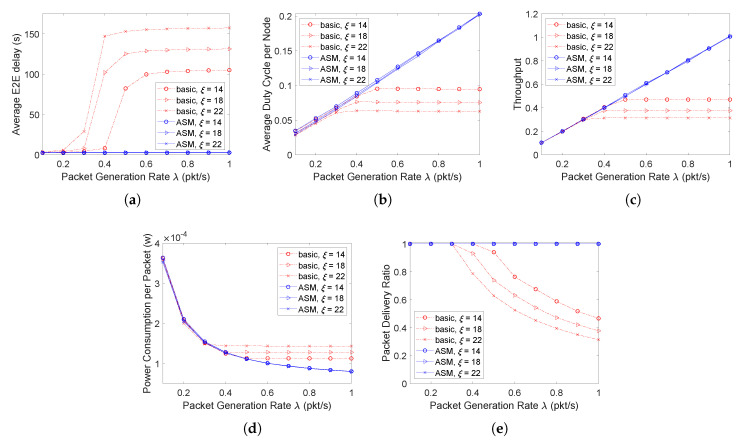
Performance evaluation in the single-chain network scenario. (**a**) Average E2E delay. (**b**) Average duty cycle. (**c**) Throughput. (**d**) Average power consumption. (**e**) Packet delivery ratio.

**Figure 8:**
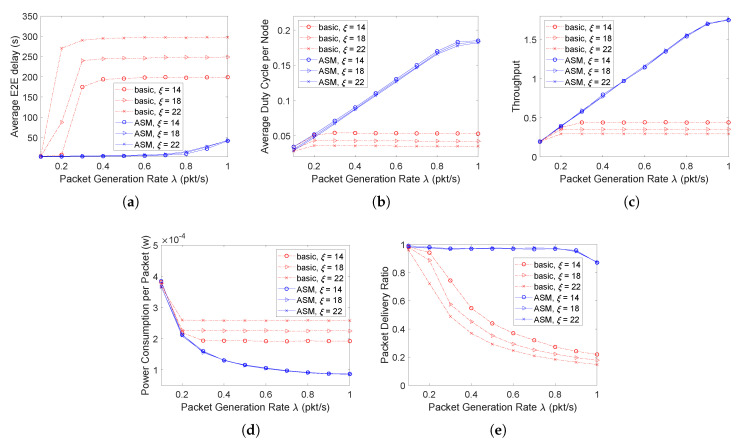
Performance evaluation in the double-chain network scenario. (**a**) Average E2E delay. (**b**) Average duty cycle. (**c**) Throughput. (**d**) Average power consumption. (**e**) Packet delivery ratio.

Based on the two chain networks, this paper then evaluates the proposed protocol in terms of average E2E delay, average duty cycle, and throughput. The comparison between the basic duty-cycling and pipelined-forwarding scheme and the D3 protocol using ASM is conducted. The simulation settings are as follows. The path length *n* is set to 10 hops. The node buffer size is set to 50 packets. Three sleep factors, 14, 18, and 22 are chosen to evaluate the effect of different sleep factors on the network performance. For D3 with ASM, according to (3), the three sleep factors respectively correspond to 3, 4, and 5 times that at most a node can wake up during its sleeping time. The packet generation rate λ for a source node varies from 0.1 to 1 packet/s. The obtained results are shown in Figure 7 and Figure 8 for the single-chain and double-chain networks, respectively.

According to the results, when the packet generation rate increases, both the E2E delay and duty cycle in the basic scheme increase first and then almost no longer change since the network traffic becomes saturated (source nodes always have packets to send). So does the network throughput—such a state is called stable state. Figure 7c shows that in the single chain network, the network becomes stable when the packet generation rates are around 0.3, 0.4, and 0.5 packet/second for the basic schemes with ξ=22,18,14, respectively. A lower packet generation rate leads to a stable network with a larger ξ. This is because as ξ increases, the network service rate decreases. Therefore, one can find that before the network becomes stable, no matter how ξ changes, the network has the same throughput under the same packet generation rate; while it is not the case after the network becomes stable, and the larger the value of ξ is, the lower the network throughput is under the same packet generation rate. In contrast, the single-chain network using ASM does not become saturated even when the packet generation rate increases to 1 packet/s, where it is saturated in the double-chain network due to the introduction of contention. Specifically, Figure 7a and Figure 8a show that the E2E delay in ASM with high data rate is much lower than that in the basic scheme. This is because in ASM, a packet can be sent out in state **S** instead of waiting for the next cycle, and the transmission in **S** can still be pipelined from the source to sink to reduce E2E delay.

Figure 7b and Figure 8b show that in the basic scheme, the network can get stable with a much shorter duty cycle (corresponding to a lower energy consumption) than that in ASM when the packet generation rate becomes higher. The energy efficiency in ASM, however, is much higher, because its average power consumption per packet is much lower (as shown in Figure 7d and Figure 8d) with a higher throughput (as shown in Figure 7c and Figure 8c) and packet delivery ratio (as shown in Figure 7e and Figure 8e). In addition, with a low packet generation rate that will not cause the network to be saturated, the duty cycle in ASM is almost the same as that in the basic scheme, which demonstrates that ASM can adaptively adjust the duty cycle according to the network traffic load.

Furthermore, for the basic scheme, as ξ increases, the E2E delay increases (as shown in Figure 7a and Figure 8a), because a larger ξ leads to a longer sleeping duration according to (2), and thus causes a packet generated during state **S** to wait for longer time to enter the next cycle. This correspondingly lead to a lower duty cycle (as shown in Figure 7b and Figure 8b), energy efficiency (as shown in Figure 7d and Figure 8d), throughput (as shown in Figure 7c and Figure 8c), and packet delivery ratio (as shown in Figure 7e and Figure 8e). While for ASM, since the sleeping time can also be utilized for data transmission on demand, ξ almost has no impact on the network performance, which further demonstrates the adaptivity of the proposed ASM mechanism to traffic load.

### 4.2. Performance Evaluation in the 2D Random Network Scenario

**Figure 9:**
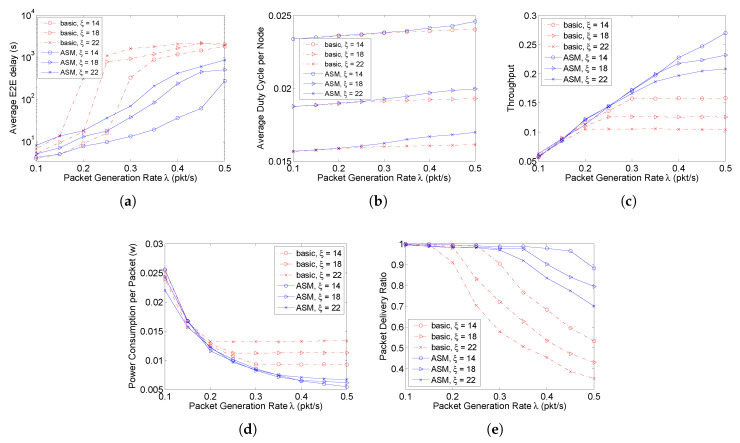
Performance evaluation in the 2D random network scenario. (**a**) Average E2E delay (7-hops). (**b**) Average duty cycle. (**c**) Throughput. (**d**) Average power consumption. (**e**) Packet delivery ratio.

In the 2D random network, the protocol is evaluated with λ varying from 0.1 to 0.5 packet/s. Since most nodes are in grade 7 (as shown in Figure 6d), in Figure 9a, it only shows the average E2E delay of the packets sent from the source nodes in grade 7. The latency increases much faster in the basic scheme than in ASM as λ increases. Similar to the chain networks, ASM in the 2D random network has a higher energy efficiency than the basic scheme, with a lower average power consumption per packet (as shown in Figure 9d), a higher throughput (as shown in Figure 9c) and packet delivery ratio (as shown in Figure 9e).

The performance difference between the chain and 2D random networks lies in that the variance of ξ also affects the performance of ASM in the random network. This is because of two reasons. First, not all nodes participate in relaying packets in the 2D random network. An idle node with a larger ξ will have a lower duty cycle (Figure 9b), which will also contribute to the final statistics. Second, the contention among nodes in the 2D network becomes more serious than that in the chain scenario due to the higher probability that the channel is sensed busy. With a larger ξ, a packet failing to be transmitted in the current cycle has to wait for a longer time to enter the next cycle, resulting in a lower energy efficiency (as shown in Figure 9d) and packet delivery ratio (as shown in Figure 9e).

## 5. Conclusions and Future Work

D3 proposed in this paper has a dynamic duty-cycling feature to achieve high energy efficiency by adaptively adjusting node duty cycle to the network traffic load, and combines MAC and routing schemes together to reduce protocol overhead and traffic congestion. In D3, the whole network is divided into grades around the sink, and staggered schedules are maintained by nodes between any two adjacent grades. A variation of the RTS/CTS handshake is utilized to forward data continuously in a pipelined fashion, largely reducing packet delivery latency to achieve real-time transmission.

For LHT in ASM introduced in Section 3.5.2, the contention among grade-one nodes for communicating with the sink node is serious, which is commonly viewed as the bottleneck of a WSN. To mitigate this issue, a hybrid reservation/contention-based (HRC) scheme is proposed based on ASM, in which the sink node assigns the sleeping slots in advance to each grade-one node (the scheme and corresponding simulation results are not shown in this paper due to the space limitation). However, the simulation results based on 2D random network show that HRC cannot outperform ASM. A possible reason lies in that the pre-assigned slots cannot be utilized efficiently due to the dynamic traffic load in the network. An on-demand slot allocation scheme is expected. In addition, the proposed schedule error correction can work seamlessly within D3, as it is designed based on the inherent characteristics of both the designed protocol and network topology. The corresponding efficacy evaluation will be conducted. We leave all of these extensions to future work.

## Figures and Tables

**Table 1:** Frame Format of RTS/CTS packet.

Fields	Comments
Grade	Grade of the node sending the packet.
Source	Address of the sending node, which is RID in this paper.
NextHop	Address of the next hop node. Set to NULL for broadcast.
StateDur	State duration right before sending the packet

**Table 2:** Frame Format of DIVISION message.

Fields	Comments
MGrade	Grade of the node sending the DIVISION message.
MState	Current state of the sending node.
MStateDur	duration of the current state right before sending the DIVISION message.

**Table 3:** Simulation Parameters.

Parameter	Value	Parameter	Value	Parameter	Value
DIFS	10 ms	SIFS	5 ms	durRTS	11 ms
durCTS	11 ms	durACK	11 ms	durDATA	43 ms
W	16	σ	1 ms	ξ	14, 18, 22
Bandwidth	10 Kbps	Tx/Rx power	0.5 W	Idle power	0.45 W
Sleep power	0.05 W	Carrier sensing range	550 m	Transmission range	250 m

## References

[1] Kandris D., Nakas C., Vomvas D., Koulouras G. (2020). Applications of Wireless Sensor Networks: An Up-to-Date Survey. Appl. Syst. Innov..

[2] GreenOrbs. http://greenorbs.org.

[3] Ayyildiz C., Erdem H.E., Dirikgil T., Dugenci O., Kocak T., Altun F., Gungor V.C. (2019). Structure health monitoring using wireless sensor networks on structural elements. Ad Hoc Netw..

[4] Abdelhafidh M., Fourati M., Fourati L.C., ben Mnaouer A., Zid M. Novel Data Preprocessing Algorithm for WSN Lifetime Maximization in Water Pipeline Monitoring System. Proceedings of the 2019 IEEE Wireless Communications and Networking Conference (WCNC).

[5] Gope P., Das A.K., Kumar N., Cheng Y. (2019). Lightweight and physically secure anonymous mutual authentication protocol for real-time data access in industrial wireless sensor networks. IEEE Trans. Ind. Inform..

[6] Ye W., Heidemann J., Estrin D. (2004). Medium access control with coordinated adaptive sleeping for wireless sensor networks. IEEE/ACM Trans. Netw..

[7] Du S., Saha A., Johnson D. RMAC: A Routing-Enhanced Duty-Cycle MAC Protocol for Wireless Sensor Networks. Proceedings of the IEEE INFOCOM 2007—26th IEEE International Conference on Computer Communications.

[8] Buettner M., Yee G.V., Anderson E., Han R. X-MAC: A short preamble MAC protocol for duty-cycled wireless sensor networks. Proceedings of the 4th International Conference on Embedded Networked Sensor Systems, SenSys 2006.

[9] Sun Y., Gurewitz O., Johnson D.B. (2008). RI-MAC: A receiver-initiated asynchronous duty cycle MAC protocol for dynamic traffic loads in wireless sensor networks. Proceedings of the 6th ACM Conference on Embedded Network Sensor Systems.

[10] Tang L., Sun Y., Gurewitz O., Johnson D. PW-MAC: An energy-efficient predictive-wakeup MAC protocol for wireless sensor networks. Proceedings of the 2011 Proceedings IEEE INFOCOM.

[11] Peng Y., Li Z., Qiao D., Zhang W. Delay-bounded MAC with minimal idle listening for sensor networks. Proceedings of the 2011 Proceedings IEEE INFOCOM.

[12] Minh N.N., Kim M.K. Reducing idle listening time in pipeline-forwarding MAC protocols of wireless sensor networks. Proceedings of the 2016 International Conference on Advanced Technologies for Communications (ATC).

[13] Ghose D., Li F.Y., Pla V. (2018). MAC protocols for wake-up radio: Principles, modeling and performance analysis. IEEE Trans. Ind. Inform..

[14] Lu G., Sadagopan N., Krishnamachari B., Goel A. Delay efficient sleep scheduling in wireless sensor networks. Proceedings of the IEEE 24th Annual Joint Conference of the IEEE Computer and Communications Societies.

[15] Cao Y., Guo S., He T. Robust multi-pipeline scheduling in low-duty-cycle wireless sensor networks. Proceedings of the 2012 Proceedings IEEE INFOCOM.

[16] Minh N.N., Kim M.K. (2017). A Cross-Layer and Enhanced Pipeline-Forwarding MAC Protocol for Wireless Sensor Network. Int. Inf. Inst. (Tokyo) Inf..

[17] Van Dam T., Langendoen K. An adaptive energy-efficient MAC protocol for wireless sensor networks. Proceedings of the 1st International Conference on Embedded Networked Sensor Systems, SenSys 2003.

[18] Lu G., Krishnamachari B., Raghavendra C.S. (2007). An adaptive energy-efficient and low-latency MAC for tree-based data gathering in sensor networks. Wirel. Commun. Mob. Comput..

[19] Lu G., Krishnamachari B., Raghavendra C. An adaptive energy-efficient and low-latency MAC for data gathering in wireless sensor networks. Proceedings of the 18th International Parallel and Distributed Processing Symposium.

[20] So H.S.W., Nguyen G., Walrand J. Practical synchronization techniques for multi-channel MAC. Proceedings of the 12th Annual International Conference On Mobile Computing and Networking.

[21] Elson J., Girod L., Estrin D. (2002). Fine-grained network time synchronization using reference broadcasts. SIGOPS Oper. Syst. Rev..

[22] Ganeriwal S., Kumar R., Srivastava M.B. Timing-sync protocol for sensor networks. Proceedings of the 1st International Conference on Embedded Networked Sensor Systems.

[23] Maróti M., Kusy B., Simon G., Lédeczi A. The flooding time synchronization protocol. Proceedings of the 2nd international Conference on Embedded networked Sensor Systems.

[24] Huang H., Yun J., Zhong Z., Kim S., He T. PSR: Practical Synchronous Rendezvous in Low-duty-cycle Wireless Networks. Proceedings of the 2013 IEEE INFOCOM.

[25] Tong F., Xie R., Shu L., Kim Y. (2011). A Cross-Layer Duty Cycle MAC Protocol Supporting a Pipeline Feature for Wireless Sensor Networks. Sensors.

[26] Hasan M.M., Karmaker A., Alam M.S., Craig A. (2019). Minimizing the Adverse Effects of Asymmetric Links: A Novel Cooperative Asynchronous MAC Protocol for Wireless Sensor Networks. Sensors.

[27] Siddiqui S., Ghani S., Khan A.A. (2017). ADP-MAC: An adaptive and dynamic polling-based MAC protocol for wireless sensor networks. IEEE Sens. J..

[28] Dutta P., Culler D. Practical asynchronous neighbor discovery and rendezvous for mobile sensing applications. Proceedings of the 6th International Conference on Embedded Networked Sensor Systems, SenSys 2008.

[29] Heimfarth T., Giacomin J.C., de Freitas E.P., Araujo G.F., de Araujo J.P. (2020). PAX-MAC: A Low Latency Anycast Protocol with Advanced Preamble. Sensors.

[30] Sahoo P.K., Pattanaik S.R., Wu S.L. (2019). A Novel Synchronous MAC Protocol for Wireless Sensor Networks with Performance Analysis. Sensors.

[31] Liu C.J., Huang P., Xiao L. (2016). TAS-MAC: A Traffic-Adaptive Synchronous MAC Protocol for Wireless Sensor Networks. ACM Trans. Sens. Netw..

[32] Tong F., Ni M., Shu L., Pan J. A pipelined-forwarding, routing-integrated and effectively-identifying MAC for large-scale WSN. Proceedings of the 2013 IEEE Global Communications Conference (GLOBECOM).

[33] Hu H., Wu Z., Li Z., Zhang X. A High-Performance Synchronous Energy-Saving Algorithm for Wireless Sensor Networks. Proceedings of the International Conference in Communications, Signal Processing, and Systems.

[34] Tong F., Tang W., Peng L., Xie R., Yang W., Kim Y. A Node-Grade Based AODV Routing Protocol for Wireless Sensor Network. Proceedings of the 2010 Second International Conference on Networks Security, Wireless Communications and Trusted Computing.

[35] Wadhwa M., Kaur K. Interference and Channel Quality Based Channel Assignment for Cognitive Radio Networks. Proceedings of the Science and Information Conference.

[36] Wu Y., Mao Z., Fahmy S., Shroff N. (2010). Constructing Maximum-Lifetime Data-Gathering Forests in Sensor Networks. IEEE/ACM Trans. Netw..

[37] Iclia V.J., Noe T.C., Carvalho M.M., Rolando M.M., Rivero-Angeles M.E., Ricardo M.M. (2018). A Selective- Awakening MAC Protocol for Energy-Efficient Data Forwarding in Linear Sensor Networks. Wirel. Commun. Mob. Comput..

[38] Singh R., Rai B.K., Bose S.K. (2019). Modeling and Performance Analysis for Pipelined-Forwarding MAC Protocols for Linear Wireless Sensor Networks. IEEE Sens. J..

[39] Nguyen K., Ji Y., Yamada S. (2013). Low Overhead MAC Protocol for Low Data Rate Wireless Sensor Networks. Int. J. Distrib. Sens. Netw..

